# Outcomes of Resistance-guided Sequential Treatment of *Mycoplasma genitalium* Infections: A Prospective Evaluation

**DOI:** 10.1093/cid/ciy477

**Published:** 2018-06-05

**Authors:** Tim R H Read, Christopher K Fairley, Gerald L Murray, Jorgen S Jensen, Jennifer Danielewski, Karen Worthington, Michelle Doyle, Elisa Mokany, Litty Tan, Eric P F Chow, Suzanne M Garland, Catriona S Bradshaw

**Affiliations:** 1 Central Clinical School, Faculty of Medicine, Nursing and Health Sciences, Monash University, Melbourne; 2 Melbourne Sexual Health Centre, Alfred Health, Carlton; 3 Murdoch Children’s Research Institute, Parkville; 4 Department of Microbiology and Infectious Diseases, Royal Women’s Hospital, Melbourne; 5 Infection and Immunity Program, Monash Biomedicine Discovery Institute; 6 Royal Children’s Hospital, Melbourne, Victoria, Australia; 7 Statens Serum Institut, Copenhagen, Denmark; 8 SpeeDx Pty Ltd, Eveleigh, New South Wales; 9 Department of Obstetrics and Gynaecology, University of Melbourne, Victoria, Australia

**Keywords:** *Mycoplasma genitalium*, urethritis, antibiotic resistance, azithromycin, sitafloxacin

## Abstract

**Background:**

Rising macrolide and quinolone resistance in *Mycoplasma genitalium* necessitate new treatment approaches. We evaluated outcomes of sequential antimicrobial therapy for *M. genitalium* guided by a macrolide-resistance assay.

**Methods:**

In mid-2016, Melbourne Sexual Health Centre switched from azithromycin to doxycycline (100 mg twice daily for 7 days) for nongonococcal urethritis, cervicitis, and proctitis. Cases were tested for *M. genitalium* and macrolide-resistance mutations (MRMs) by polymerase chain reaction. Directly after doxycycline, MRM-negative infections received 2.5 g azithromycin (1 g, then 500 mg daily for 3 days), and MRM-positive infections received sitafloxacin (100 mg twice daily for 7 days). Assessment of test of cure and reinfection risk occurred 14–90 days after the second antibiotic.

**Results:**

Of 244 evaluable *M. genitalium* infections (52 women, 68 heterosexual men, 124 men who have sex with men) diagnosed from 20 June 2016 to 15 May 2017, MRMs were detected in 167 (68.4% [95% confidence interval {CI}, 62.2%–74.2%]). Treatment with doxycycline decreased bacterial load by a mean 2.60 log_10_ (n = 56; *P* < .0001). Microbiologic cure occurred in 73 of 77 MRM-negative infections (94.8% [95% CI, 87.2%–98.6%]) and in 154 of 167 MRM-positive infections (92.2% [95% CI, 87.1%–95.8%]). Selection of macrolide resistance occurred in only 2 of 76 (2.6% [95% CI, .3%–9.2%]) macrolide-susceptible infections.

**Conclusions:**

In the context of high levels of antimicrobial resistance, switching from azithromycin to doxycycline for presumptive treatment of *M. genitalium*, followed by resistance-guided therapy, cured ≥92% of infections, with infrequent selection of macrolide resistance.

## Teaser

Treating *Mycoplasma genitalium* with a sequence of first doxycycline to reduce bacterial load, and then using a resistance assay to choose either high-dose azithromycin or sitafloxacin, cured >92% of infections in a population with high levels of antibiotic resistance.

**(See the Major Article by Braun et al on pages 569-76 and Editorial commentary by Sulkowski on pages 577-9.)**

*Mycoplasma genitalium* is a sexually transmitted cause of nongonococcal urethritis (NGU) and is associated with cervicitis, pelvic inflammatory disease (PID), and poor obstetric outcomes [1, 2]. Recommended first-line treatment for *M. genitalium* has been azithromycin, but this is now known to fail in at least 10% of susceptible infections, leading to selection (posttreatment detection) of strains with macrolide-resistance mutations (MRMs) at positions 2058 or 2059 in the 23S ribosomal RNA (rRNA) gene [3, 4]. We use the term “selection,” but it is unknown to what extent this represents the selection of resistance emerging during treatment vs selection of minority populations with preexisting resistance mutations. Macrolide resistance is now reported in >50% of diagnosed infections in many countries, having been uncommon a decade ago [5–11].

Culturing *M. genitalium* is difficult and performed in few centers worldwide, limiting our understanding of its antibiotic susceptibilities. *Mycoplasma genitalium* has no cell wall, limiting treatment choice to macrolides or later-generation fluoroquinolones [3, 12]. The streptogramin pristinamycin has recently been shown to cure only 75% of macrolide-resistant infections [13]. Furthermore, a meta-analysis has shown a decline in cure for moxifloxacin from 100% in studies prior to 2010, to 89% in studies from 2010 onward [14]. ParC fluoroquinolone resistance mutations, which reduce the efficacy of moxifloxacin, were recently detected in 14%–15% of Australian cases, 27%–40% of cases in human immunodeficiency virus–infected men in the United States, and 47% of cases in Japan [8, 15–17]. These data suggest that continuing to treat *M. genitalium* with single agents will increase the prevalence of resistance and reduce remaining treatment options [12].

In response, European, British, and Australian treatment guidelines have recently recommended azithromycin be replaced as initial treatment for NGU, with doxycycline 100 mg twice daily for 7 days [18, 19]. Doxycycline is highly effective for *Chlamydia trachomatis* but only cures about a third of *M. genitalium* infections; however, it does not appear to select further identifiable resistance in treatment failures. Furthermore, studies of extended azithromycin treatment of *M. genitalium* show better outcomes for infections that were previously treated with doxycycline than those that were not, suggesting that doxycycline may have influenced the outcome of subsequent macrolide treatment [3, 4]. Because several studies have shown that *M. genitalium* infections with lower loads were more likely to be cured, it may be that doxycycline lowered bacterial load, rendering *M. genitalium* more susceptible to a subsequent macrolide [11, 13, 20].

In 2016, in response to macrolide resistance exceeding 50%, (20% of these also quinolone resistant) [11, 17] and diminishing treatment options, the Melbourne Sexual Health Centre (MSHC) introduced a 3-step approach to treatment of *M. genitalium*. First, azithromycin was replaced with doxycycline (100 mg twice daily for 7 days) for the treatment of NGU, proctitis, and cervicitis. Second, patients were tested with a combined diagnostic/resistance assay that detected *M. genitalium* and the 5 main MRMs in 23S rRNA (ResistancePlus MG, SpeeDx, Australia) [21]. Third, treatment for *M. genitalium* was guided by the macrolide-resistance result. Macrolide-susceptible infections received a higher extended dose of azithromycin of 2.5 g (1 g followed by 500 mg daily for a total of 4 days), and macrolide-resistant infections received sitafloxacin 100 mg twice daily for 7 days. This dose of azithromycin was selected because, although some studies suggest better outcomes with 1.5 g compared with 1 g [4], we found no significant improvement in *M. genitalium* cure with 1.5 g compared to the 1-g dose and postulated that the dose increase was insufficient to consistently increase proportions cured [11]. Sitafloxacin was used instead of moxifloxacin because in vitro data show lower mean inhibitory concentrations than moxifloxacin in moxifloxacin-susceptible isolates, and sitafloxacin retains activity in some strains with moxifloxacin resistance (Jorgen Jensen, Statens Serum Institut, Copenhagen, personal communication, June 2015). Patients diagnosed with *M. genitalium* without NGU, proctitis, or cervicitis, such as sexual contacts of individuals infected with *M. genitalium*, were also pretreated with a week of doxycycline. We aimed to increase the proportion of *M. genitalium* infections cured above 90% and to reduce selection of macrolide resistance.

## METHODS

This was a prospective evaluation of patients treated by resistance-guided therapy for *M. genitalium* infections at MSHC from 20 June 2016 to 15 May 2017. MSHC is the only public clinic treating sexually transmitted infections (STIs) in Melbourne, a city of 4.5 million. Cases were evaluated if they did not receive azithromycin initially, they received doxycycline 100 mg twice daily for 7 days, were diagnosed with *M. genitalium* using a diagnostic-resistance polymerase chain reaction (PCR) assay (ResistancePlus MG, SpeeDx Pty Ltd, Australia), and if treatment was based on their macrolide-resistance result. Those with no detectable MRMs received azithromycin 2.5 g (1 g followed by 500 mg daily for a total of 4 days), and those with detectable MRMs received sitafloxacin 100 mg twice daily for 7 days.

Patients were asked to return for a test of cure using the same assay 21–28 days after starting sitafloxacin or azithromycin (28–35 days after commencing doxycycline). Nurses made at least 2 attempts to contact those who failed to attend. Clinicians performing a test of cure used a template to enter data in the electronic medical record. The template listed key variables, including persistence of symptoms, adherence to each antibiotic, adverse events, and posttreatment sexual exposure to new or continuing partners, and whether those partners were treated.

A substudy to measure the impact of doxycycline on organism load was performed in 56 patients with *M. genitalium* urethritis. When patients returned for their second antibiotic, a subset of patients were asked for a urine sample, and the number of doses of doxycycline taken was recorded. These urine samples were stored frozen at –80°C and at the end of the study were tested simultaneously by quantitative PCR for *M. genitalium* load alongside the original diagnostic sample. To examine selection of macrolide resistance, Sanger sequencing was also performed on pre- and posttreatment samples that had been classified as susceptible by the resistance PCR assay, treated with azithromycin, and failed, to determine if selection of macrolide resistance had occurred, as described previously [7].

Patients were included in the study if (1) treatment followed the protocol; (2) they returned for a test of cure at 14–90 days after starting their second antibiotic; and (3) they did not report ongoing condomless sex with a pretreatment partner, unless this partner had also completed treatment. Patients not meeting these criteria were excluded, regardless of test-of-cure result, to prevent bias.

Test-of-cure results were stratified by posttreatment reinfection risk, based on responses to questions on the clinical template. Reinfection risk was categorized as follows: no sex since treatment, sex with 100% condom use, any condomless sex with a fully treated partner, any condomless sex with a new partner, or any condomless sex with a partner who had not completed treatment. The association with each category and positive test of cure was examined by univariate logistic regression. Pretreatment and posttreatment *M. genitalium* bacterial load data among men with NGU were log_10_ transformed and means were compared by paired *t* test. Proportions of men who have sex with men (MSM) and heterosexuals with macrolide resistance were compared by χ^2^ test. The 95% confidence intervals (CIs) of proportions were calculated by exact methods. The Alfred Hospital Ethics Committee approved this study (approval number 232/16).

## RESULTS

**Figure 1.**
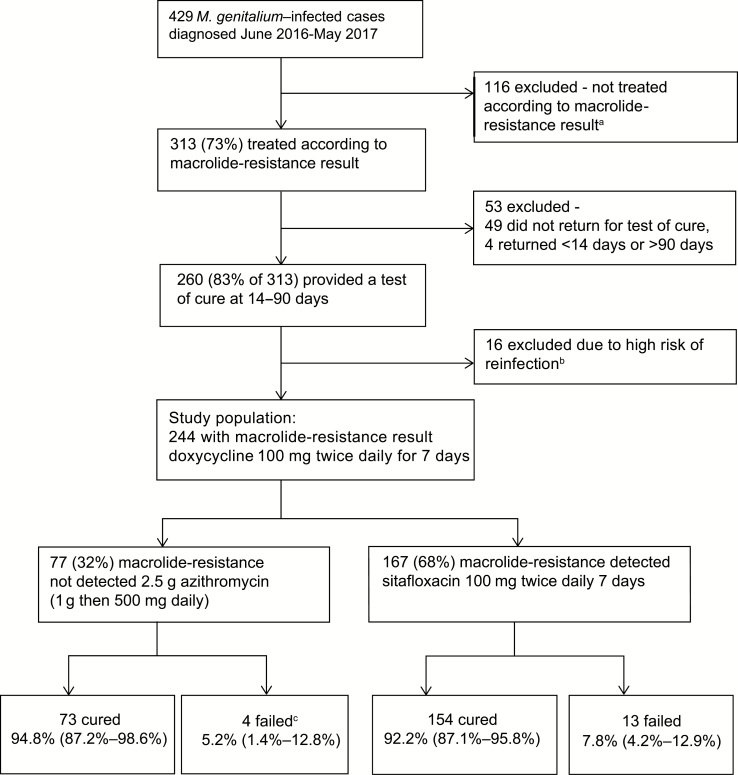
Selection of cases and outcomes of resistance-guided sequential treatment of *Mycoplasma genitalium* infections. Bottom row: data in parentheses indicate the 95% confidence interval. ^a^Reasons why 116 patients were not treated according to the resistance result and were excluded: women treated for pelvic inflammatory disease (n = 33), treatment initiated in the community (n = 23), patients given single-dose azithromycin at diagnosis, mostly with ceftriaxone for gonorrhea (n = 22), individuals who did not return for treatment (n = 16), patients given pristinamycin because quinolones were contraindicated (n = 12), other variations in dose or medication choice (n = 10). ^b^These individuals reported condomless sex with an untreated sexual partner. ^c^Sanger sequencing of pretreatment samples from these 4 individuals identified macrolide-resistance mutations (MRMs) in 1 sample. MRMs were detected in 2 of the remaining 3 samples, meaning MRMs emerged during treatment in 2 of 76 cases.

Of 429 *M. genitalium* infections diagnosed during the study period, 313 (73%) were treated according to the resistance-guided protocol (Figure 1). The commonest reasons for not following the protocol were azithromycin treatment at diagnosis (eg, coexisting gonorrhea) or contraindications to fluoroquinolones. Of 313 treated according to the protocol, 49 did not return and 4 returned outside the 14- to 90-day follow-up interval, leaving 260 (83.1%) in whom treatment outcome could be determined. A further 16 were excluded because they reported condomless sex with untreated or incompletely treated partners. This exclusion was supported by the strong association with a positive test of cure (odds ratio, 6.65 [95% CI, 1.85–23.84]; Table 1).

**Table 1.** Results of Test of Cure by Patient-reported Reinfection Risk After Commencing Treatment

Data on Reinfection Risk (N = 240^a^)	No. (% [95% CI])	Positive Test of Cure, No. (%)	Odds Ratio (95% CI)	*P* Value
No sex	125 (52.1 [45.6–58.6])	8 (6.4)	reference	
Condom use 100% with all partners	60 (25.0 [19.7–31.0])	7 (11.7)	1.93 (.67–5.60)	.226
Any condomless sex with a fully treated partner	14 (5.8 [3.2–9.6])	1 (7.2)	1.13 (.13–9.72)	.915
Any condomless sex with a new partner	25 (10.4 [6.9–15.0])	1 (4.0)	0.61 (.07–5.10)	.648
Any condomless sex with an incompletely treated partner^b^	16 (6.7 [3.9–10.6])	5 (31.3)	6.65 (1.85–23.84)	.004

Abbreviation: CI, confidence interval.

^a^Twenty patients (7.7%) with no reinfection risk data were excluded from this analysis but were retained in the analysis of treatment outcomes.

^b^These 16 patients were excluded from the analysis of treatment outcomes but shown here to highlight the high odds of failure in individuals reporting condomless sex with an untreated partner, compared with the other categories. Men who have sex with men status was not associated with positive test of cure (*P* = .903).

Characteristics of the remaining 244 participants are shown in Table 2. The median time to test of cure, from the start of the second antibiotic, was 28 days (interquartile range [IQR], 22–35 days) and patients commenced their second antibiotic a median of 7 days (mean, 8.5 days) from initiation of doxycycline. The 2 most common presentations were NGU (45%) and being a sexual contact of a person with *M. genitalium* infection (24%); 75% of all participants reported symptoms. Macrolide-resistance mutations were detected in 167 of 244 (68.4% [95% CI, 62.2%–74.2%]) infections, and were more common in MSM (87.1% [95% CI, 79.9%–92.0%]) than in heterosexual men and women (49.2% [95% CI, 40.3%–58.1%]) (*P* < .0001).

**Table 2.** Characteristics of the Population Studied for Outcomes of *Mycoplasma genitalium* Treatment

Characteristic	Macrolide Susceptible (n = 77)	Macrolide Resistant (n = 167)	Total (N = 244)
Time to test of cure^a^, d, median (IQR)	29 (25–41)	27 (22–32)	28 (22–35)
Age, y, median (IQR)	26.0 (22.9–30.9)	28.7 (25.6–34.4)	27.9 (24.5–33.0)
Sex/sexuality^b^			
Female	29 (37.7)	23 (13.8)	52 (21.3)
Male, heterosexual	32 (41.6)	36 (21.6)	68 (27.9)
MSM	16 (20.8)	108 (64.7)	124 (50.8)
Site of detection^c^			
Cervix/vagina	24 (31.1)	21 (12.6)	45 (18.4)
Urine	49 (63.6)	105 (62.9)	154 (63.1)
Rectum^d^	4 (5.2)	41 (24.6)	45 (18.4)
HIV serostatus			
Negative	62 (80.5)	141 (84.4)	203 (83.2)
Untested	15 (19.5)	10 (6.0)	25 (10.3)
Positive	0	16 (9.58)	16 (6.56)
Asymptomatic^c^	18 (23.4)	43 (25.8)	61 (25.0)
Symptomatic	59 (76.6)	124 (74.3)	183 (75.0)
Clinical diagnosis			
Nongonococcal urethritis	31 (40.3)	78 (46.7)	109 (44.7)
Contact of *Mycoplasma genitalium*	21 (27.3)	38 (22.8)	59 (24.2)
Other^e^	5 (6.5)	28 (16.8)	33 (13.5)
Vaginal discharge/bleeding	13 (16.9)	7 (4.2)	20 (8.2)
Proctitis	3 (3.9)	12 (7.2)	15 (6.2)
Cervicitis/PID	4 (5.2)	4 (2.4)	8 (3.3)

Data are presented as No. (%) unless otherwise indicated.

Abbreviations: HIV, human immunodeficiency virus; IQR, interquartile range; MSM, men who have sex with men; PID, pelvic inflammatory disease.

^a^Time to test of cure defined as days from the start of the second (resistance-guided) antibiotic.

^b^Female includes 5 women who have sex with women. MSM includes 3 transgender women.

^c^No. and proportion with macrolide-resistance mutations were: urine, 105 (68.2%); rectum, 41 (91.1%); cervix/vagina, 21 (46.7%); asymptomatic, 43 (70.5%); symptomatic, 124 (67.8%).

^d^Includes 4 multisite infections.

^e^Other diagnoses included contact of chlamydia or gonorrhea (n = 9), urethral gonorrhea (n = 3), pelvic pain not diagnosed as PID (n = 3), dyspareunia (n = 2), dysuria (n = 2), anal itch/discharge (n = 4), vaginal candidiasis (n = 3), bacterial vaginosis (n = 2), balanitis (n = 2), pain with defecation (n = 2), epididymitis (n = 2).

### Treatment Outcomes

Of the 77 macrolide-susceptible cases receiving doxycycline then 2.5 g azithromycin, 73 were microbiologically cured (94.8% [95% CI, 87.2%–98.6%]) and 4 had positive tests of cure (5.2% [95% CI, 1.4%–12.8%]). Macrolide-resistance mutations were detected in the posttreatment samples of 3 of the 4 failures by the resistance PCR assay. Sequencing of the 23S rRNA gene in the posttreatment samples confirmed that 3 were macrolide resistant and 1 was wild type. This wild type was detected 40 days posttreatment in a woman reporting no missed doses and full condom use with a new partner. Subsequent sequencing of the 23S rRNA gene in the pretreatment samples from these 4 individuals detected MRMs in 1 sample (with a posttreatment MRM detected) that had not been detected by the resistance PCR assay. Therefore, selection of MRM was observed in only 2 of the azithromycin failures (2/76 macrolide-susceptible infections; 2.6% [95% CI, .3%–9.2%]). Of the 167 macrolide-resistant cases treated with doxycycline followed by sitafloxacin, 154 were microbiologically cured (92.2% [95% CI, 87.1%–95.8%]), and 13 had positive tests of cure (7.8% [95% CI, 4.2%–12.9%]) (Figure 1). Overall, 227 of 244 (93.0% [95% CI, 89.1%–95.9%]) were cured. The proportion cured did not differ between sexual risk groups, sites of infection, and symptom status, lying between 91.1% and 95.1% in these groups.

### Adherence and Adverse Events

Adherence data were recorded by clinicians in 217 of 244 (88.9%) patients for doxycycline, 152 of 167 (91.0%) patients for sitafloxacin, and 63 of 77 (81.8%) patients for azithromycin. Self-reported adherence was high with 89.9%, 90.8%, and 100%, respectively, reporting taking all doses of prescribed antimicrobials (Table 3). Adverse event data were recorded in 224 of 244 (91.8%) patients and, of these, the proportions reporting no adverse events were 86.6% for doxycycline, 91.4% for azithromycin, and 80.5% for sitafloxacin. Commonly reported adverse events were as follows: for doxycycline: nausea (5.4%) and diarrhea (4.9%); for azithromycin: nausea (5.7%); and for sitafloxacin: diarrhea (11.7%) and tendon/joint pain (5.2%) (Table 3). The only reported adverse event leading to cessation of sitafloxacin was 1 case of patchy hypoesthesia on the limbs and face of a man, which did not appear to be peripheral neuropathy and resolved after 3 weeks.

**Table 3.** Self-reported Adherence and Adverse Events^a^ Associated With Antibiotics Used in the Sequential Treatment Regimens

	Doxycycline^b^	Azithromycin	Sitafloxacin^c^
Adherence	n = 217^d^	n = 63	n = 152
Took all doses	195 (89.9)	63 (100)	138 (90.8)
Missed 1–4 doses	18 (8.3)	0	10 (6.6)
Missed >4 doses	4 (1.8)	0	4 (2.6)
Adverse events	n = 224^d^	n = 70	n = 154
None reported	194 (86.6)	64 (91.4)	124 (80.5)
Nausea	12 (5.4)	4 (5.7)	5 (3.2)
Vomiting	2 (0.9)	0	1 (0.6)
Diarrhea	11 (4.9)	2 (2.9)	18 (11.7)
Rash/sunburn^e^	6 (2.7)	0	1 (0.6)
Tendon/joint pain	0	0	8 (5.2)
Headache or dizziness	3 (1.3)	0	2 (1.3)

Data are presented as No. (%) unless otherwise indicated.

^a^Adverse events were only grade 1 (no interference with daily activities and no treatment required) or grade 2 (minor interference with daily activities or required minor treatment).

^b^Grade 2 events were vomiting (n = 1), diarrhea (n = 2), rash (n = 1). All others were grade 1.

^c^Grade 2 events were diarrhea (n = 5), tendinitis (n = 1), self-limiting hypoesthesia (n = 1). All others were grade 1.

^d^The total receiving each drug was doxycycline, n = 244; azithromycin, n = 77; sitafloxacin, n = 167. Individuals with no data on adherence or adverse events were excluded from those analyses.

^e^Sunburn (n = 5) and rash (n = 1).

### Bacterial Load

**Figure 2.**
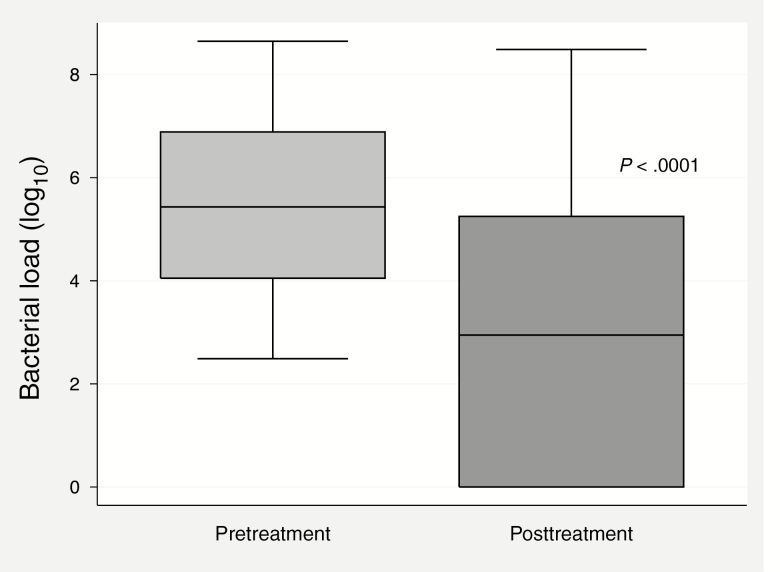
Bacterial load (log_10_) of *Mycoplasma genitalium* in urine samples before and after doxycycline 100 mg twice daily for 7 days (n = 56).

*Mycoplasma genitalium* load in urine, before and during or immediately after doxycycline, was measured in 56 men with urethritis. At the time of sample collection, men had taken a median of 13 of 14 doses (IQR, 11–14 doses). *Mycoplasma genitalium* load was undetectable in 22 (39%) men, reduced but detectable in 28 (50%) men, and increased in 6 (11%) men. Mean bacterial load declined by 2.60 log_10_ from pretreatment levels (*P* < .0001; Figure 2).

## DISCUSSION

This study demonstrates that >92% of anogenital *M. genitalium* infections can be cured in a population where two-thirds of cases are macrolide resistant and 20% of macrolide-resistant cases are likely quinolone resistant [15, 17]. This was achieved by pretreating with doxycycline and selecting a second antimicrobial with a macrolide-resistance assay. Selection of macrolide resistance occurred in <3% of macrolide-susceptible infections treated with sequential therapy. Replacing azithromycin with doxycycline for initial treatment of STI syndromes has the dual advantage of reducing overall use of azithromycin and reducing *M. genitalium* load. We hypothesize that reduction in bacterial load before commencing the second antimicrobial contributed to the high proportion cured and reduced the rate of selected macrolide resistance. In our clinic population, adherence to this regimen was high and antimicrobials were generally well tolerated, but fourth-generation fluoroquinolones are expensive, and almost 20% of patients taking sitafloxacin reported some adverse event. Further research is required to determine if all components of this treatment protocol (ie, doxycycline pretreatment, choice and dosage of antibiotics) are necessary to achieve these high levels of cure.

The outcomes in the doxycycline-azithromycin treatment arm are better than in contemporary studies of azithromycin treatment of unselected *M. genitalium*. A meta-analysis of 21 studies of single-dose 1 g azithromycin showed that proportions cured fell from 85% before 2009 to 67% afterward, consistent with a rising prevalence of resistance [22]. In the 3 randomized trials comparing doxycycline and single-dose azithromycin for NGU, proportions with *M. genitalium* cured following azithromycin declined from 87% to 67% to 40% in studies published in 2009, 2011, and 2013, respectively [23–25]. A meta-analysis by Horner et al found that single-dose 1 g azithromycin failed in 13.9% of 353 cases known to be macrolide susceptible, resulting in the selection of macrolide resistance in 12.0% (95% CI, 7.1%–16.9%) [4]. With doxycycline pretreatment and an increased dose of azithromycin, we observed selection of macrolide resistance in only 2 of 76 macrolide-susceptible cases (2.6% [95% CI, .3%–9.2%]). While Horner et al’s meta-analysis reported selected resistance in a similar proportion of cases (3/82 [3.7%]) treated with azithromycin 1.5 g over 5 days [4], in a recent study at our center, 4 of 34 (11.8%) cases treated with the 1.5-g azithromycin regimen developed selected resistance [11]. Horner et al’s meta-analysis excluded 56 cases treated with 1.5 g azithromycin that had previously received doxycycline and, interestingly, there were no cases of selected resistance among these [4]. As the proportion of cases with selected resistance in our study (2.6%) is not significantly lower than any of these estimates, we cannot determine to what extent the low rate in our study is due to the doxycycline vs the increased dose and duration of azithromycin.

Outcomes in the doxycycline-azithromycin arm also depend upon the sensitivity of the PCR test for detecting resistance mutations. In this study, 1 of the 4 azithromycin treatment failures was found to be macrolide resistant when the pretreatment sample was sequenced. Therefore, false-negative results from the resistance PCR assay may account for some failures of azithromycin in cases thought to be macrolide susceptible. Published evaluations of the ResistancePlus assay have varied in their methods, likely explaining some variation in reported sensitivity, but false-negative results do occur [21, 26, 27]. Clinicians should be aware of this limitation, particularly when confronted by unexpectedly macrolide-susceptible cases such as after azithromycin treatment failure.

The outcomes in the doxycycline-sitafloxacin arm appear better than expected given that ParC fluoroquinolone-associated resistance mutations were found in 12 of 60 (20% [95% CI, 8.4%–20.4%]) of all macrolide-resistant *M. genitalium* infections at MSHC in 2012–2013 [17]. Four years later we would therefore expect moxifloxacin to fail in at least 20% of patients in the macrolide-resistant group. However, the sequential doxycycline-sitafloxacin regimen failed in a significantly lower proportion 7.8% (95% CI, 4.2%–12.9%) of infections (*P* = .010). Sequencing is under way to determine the prevalence of ParC and GyrA resistance mutations in this group and to identify mutations associated with sitafloxacin failure.

It appears likely that doxycycline pretreatment contributed to the relatively high efficacy (92%–95%) of antibiotics in this study population. Lower bacterial load predicts treatment success with macrolides and pristinamycin, and we observed a significant 2.6-log fall in *M. genitalium* load among men with NGU after 7 days of doxycycline [11, 13, 20]. Further research with controls is required to determine the impact of doxycycline pretreatment on fluoroquinolone failure and whether sequential or simultaneous treatment is more effective.

There are important limitations in the ability of this study to determine treatment efficacy. First, the intervention has multiple components, which were not implemented separately. We cannot therefore disentangle the effect of doxycycline premacrolide from the higher dose of azithromycin in improving cure and reducing selection of macrolide resistance. Further research is required to establish the contribution of each component to improved cure. However, given current unacceptable failure rates with recommended regimens, this approach provides an interim measure to combine with monitoring of treatment outcomes until further data become available. We also cannot determine if sitafloxacin is superior to moxifloxacin in this population, as suggested by in vitro data. Second, there is no control group in this study. However, the controlled trials of treatments for PID and NGU were not adequately powered to assess efficacy for *M. genitalium* because it causes a minority of cases in these syndromes. Randomized trials of treatments specific for *M. genitalium* have not been conducted, in part because diagnosis is usually delayed until after syndromic therapy is commenced. The absence of controls means that it is even more important to exclude individuals with a high risk of reinfection from the analysis of treatment efficacy. While self-report is not considered highly reliable, we observed increased treatment failures only in the group with the highest reported risk of reinfection, suggesting that this is a valid measure. All patients reporting this level of risk were excluded, removing any bias toward a favorable result. Finally, the effect of doxycycline on bacterial load was only measured in men with NGU and it may not be the same in other sites of infection or in asymptomatic infection.

Replacing azithromycin with doxycycline for the initial treatment of STI syndromes, and increasing the dose of azithromycin, appears to increase proportions cured and to reduce selection of macrolide-resistant mutants. The increased dose of azithromycin was well tolerated. Furthermore, resistance-guided treatment enables continued use of the cheaper and safer azithromycin for macrolide-susceptible infections. As the prevalence of macrolide resistance rises, there are fewer opportunities to use azithromycin. However, this study indicates the potential benefits of resistance-guided treatment, and future assays detecting quinolone resistance will refine treatment approaches and improve outcomes [28]. Studies of sequential resistance-guided therapy using doxycycline followed by moxifloxacin are under way. Point-of-care assays for *M. genitalium* with resistance outputs are also in development and, when combined with point-of-care testing for chlamydia and gonorrhea, will enable a shift from syndromic management to resistance-guided etiologic treatment [28]. These assays will allow randomization for studies to occur before treatment commences, facilitating controlled trials of *M. genitalium* treatments. While resistance-guided treatment currently cures ≥92% of *M. genitalium* infections, this requires a later-generation fluoroquinolone with the cost and potential side effects this entails. These risks should be considered before patients are tested for *M. genitalium.*

## Notes

***Acknowledgments.*** We thank the staff of Melbourne Sexual Health Centre (MSHC) for collecting the clinical data for this study.

***Financial support.*** This work was supported by early career research fellowships from the National Health and Medical Research Council (award numbers 1091536 to T. R. H. R. and 1091226 to E. P. F. C.).

***Potential conflicts of interest.*** K. W. and M. D. are research staff receiving partial salary support from SpeeDx Pty Ltd. E. M. and L. T. are employed by SpeeDx. J. S. J. has received speaker’s fees from Hologic; serves on a scientific advisory board for Roche Molecular Systems; and has performed contract work for SpeeDx, Diagenode, NYtor, Cempra Pharmaceuticals, Nabriva, C10 Pharma, and Angelini. J. S. J.’s employer, the Statens Serum Institut has received remuneration for contract work from SpeeDx, Hologic, NYtor, Diagenode, Nabriva, GSK, and Angelini. S. M. G. has received grants from the Department of Business, the Australian Federal Government, Merck, GSK, CSL, the Commonwealth Department of Health, and Merck, and nonfinancial support from Merck; and has delivered lectures and received speaking fees from MSD and personal fees from SPMSD. G. L. M. has received grants from the Australian Federal Government (Department of Business and Innovation), and grants and nonfinancial support from SpeeDx. All other authors are employed by the Royal Women’s Hospital or MSHC, which both receive research funding from SpeeDx. Hologic has also provided test kits to MSHC. All authors have submitted the ICMJE Form for Disclosure of Potential Conflicts of Interest. Conflicts that the editors consider relevant to the content of the manuscript have been disclosed.
